# Role of Exosomes in Cancer-Related Cognitive Impairment

**DOI:** 10.3390/ijms21082755

**Published:** 2020-04-15

**Authors:** Yong Qin Koh, Chia Jie Tan, Yi Long Toh, Siu Kwan Sze, Han Kiat Ho, Charles L. Limoli, Alexandre Chan

**Affiliations:** 1 Department of Pharmacy, Faculty of Science, National University of Singapore, Singapore 119077, Singapore; 2 School of Biological Sciences, Nanyang Technological University, 60 Nanyang Drive, Singapore 637551, Singapore; 3 Department of Radiation Oncology, University of California, Irvine, CA 92697-2695, USA; 4 Department of Clinical Pharmacy Practice, University of California, Irvine, CA 92697, USA

**Keywords:** exosomes, cancer, chemotherapy, cognitive impairment, cell communication

## Abstract

A decline in cognitive function following cancer treatment is one of the most commonly reported post-treatment symptoms among patients with cancer and those in remission, and include memory, processing speed, and executive function. A clear understanding of cognitive impairment as a result of cancer and its therapy can be obtained by delineating structural and functional changes using brain imaging studies and neurocognitive assessments. There is also a need to determine the underlying mechanisms and pathways that impact the brain and affect cognitive functioning in cancer survivors. Exosomes are small cell-derived vesicles formed by the inward budding of multivesicular bodies, and are released into the extracellular environment via an exocytic pathway. Growing evidence suggests that exosomes contribute to various physiological and pathological conditions, including neurological processes such as synaptic plasticity, neuronal stress response, cell-to-cell communication, and neurogenesis. In this review, we summarize the relationship between exosomes and cancer-related cognitive impairment. Unraveling exosomes’ actions and effects on the microenvironment of the brain, which impacts cognitive functioning, is critical for the development of exosome-based therapeutics for cancer-related cognitive impairment.

## 1. Introduction

The uncontrolled division and cellular changes that lead to abnormal cell growth and metastasis are defining hallmarks of cancer [1]. Cancer cells do not respond appropriately to the signals that control normal cellular behavior, with the potential to spread throughout the body and invade normal tissues and organs. The conventional therapies for cancer, such as chemotherapy and radiation therapy, aim to ablate cancer cells through a variety of mechanisms that include mitotic catastrophe, necroptosis, autophagy, and apoptosis [2,3,4]. However, these treatments can lead to many short- and long-term side effects including cachexia, fatigue, neuropathy, and cognitive impairment, several of which impair patients’ quality of life and ability to function. Given the increased number of individuals who survive after a cancer diagnosis, one of the key concerns arising among cancer survivors is a decline in cognitive performance [5,6].

Cognitive impairments that occur following cancer diagnosis and treatment are collectively known as cancer-related cognitive impairment (CRCI) [7]. In recent years, a fairly consistent body of research has reported on the clinical complaints related to CRCI in both pediatric and adult cancer survivors [8,9]. CRCI is characterized as deficits in several cognitive domains including memory, attention, learning, and executive function [10]. CRCI adversely affects cancer patients and survivors, causing distress and reduced quality of life, and impacts many facets of their daily lives [11]. CRCI is complicated by many factors including the direct effects of cancer, genetic predisposition (e.g., carrier of apolipoprotein E type epsilon 4 allele [12] and brain-derived neurotrophic factor Met allele [13]), comorbidities independent of the actual disease, and/or treatments or combinations of treatments administered for the disease [14].

Efforts to better understand cancer- and cancer-therapy-associated cognitive impairment have relied on methods ranging from cognitive functioning assessment to imaging technologies on brain structure and function (e.g., magnetic resonance imaging scan). However, these approaches may be limited to help us understand the etiology and pathophysiology of cognitive dysfunction. The emerging application of the use of liquid biopsies (e.g., plasma and cerebrospinal fluid) as a strategy for biomarker discovery to detect, assess and monitor CRCI is fast-expanding and evolving [15,16]. In published literature, studies have revealed that genetics and neurobiological and immunological mechanisms may be involved in the biogenesis and development of CRCI. Preclinical studies have also provided insights into the pathophysiological mechanisms underlying CRCI, including neurotoxicity and inflammatory factors, which were shown to be responsible for CRCI [17]. Nevertheless, the underlying mechanisms of CRCI have yet to be established. While the role of exosomes in cancer has been well-documented [18,19,20], the role of cancer exosomes and their ability to interact with the nervous system to modulate neurological processes such as neuronal functioning and stress response have attracted considerable attention.

Exosomes, as a distinct class of extracellular vesicles with spherical morphology and size ranging from 30–150 nm, were found to influence physiological and pathological conditions, such as immune homeostasis, pregnancy, infectious diseases, cancer, and neurological disorders [15,21]. Exosomes are endocytic vesicles formed by the inward budding of multivesicular bodies. Their fusion with the cellular plasma membrane results in their extracellular release. Exosome origin determines cell targeting and the transfer of its content into the targeted cell. The composition of exosomes is influenced by a variety of factors, especially dependent on the origin of the cells from which they are released [22]. Depending on the nature of exosomes, they may have many roles in different physiological and pathological settings, with beneficial or detrimental activity [23,24]. 

Exosomes have emerged as important mediators of intercellular communication with the ability to shuttle bioactive molecules (e.g., microRNAs [miRNAs] and proteins) between neighboring and distant cells, modulating the biological activities such as immune response, cell proliferation, and cell signaling pathways of the recipient cells. In this review, we highlight the role of exosomes in intercellular signaling in the nervous system and discuss the existing evidence on the relationships of exosomes in cancer-related symptoms, with a focus on CRCI and its potential value in CRCI research.

## 2. Methods

Published literature based on a PubMed and ScienceDirect search using a combination of the terms [extracellular vesicles] or [vesicles] or [exosom*] and [cognit*] or [impair and [cancer] or [metast*] and [therapy] or [treat*] in their title or abstract were evaluated. Included studies fulfilled guidelines recommended for extracellular vesicle characterization (e.g., the report of at least one extracellular vesicle marker of transmembrane and cytosolic proteins) as well as experimental procedures involving their separation and isolation [25,26,27].

## 3. Exosomes and the Nervous System

The nervous system, made up of a highly interconnected neuronal network, responds to internal and external stimuli, and is responsible for relaying sensory information and coordinating bodily function. Neurons are nerve cells within the nervous system that serve to sense, process, and transmit signals between different parts of the body. Neurons communicate with each other through electrical signals (e.g., action potentials) and chemical mediators (e.g., neurotransmitters) [28]. In recent years, emerging evidence has indicated that exosomes mediate inter-neuronal communication and play a critical role in neuronal physiology and pathology [29]. The dynamics, activities, and signaling among neurons and/or non-neuronal cells, through either electrical signals, chemical activities, or exosomes are believed to impact our perceptions, thoughts, and behaviors [30].

### 3.1. Exosomes and Neuronal Communication

**Figure 1:**
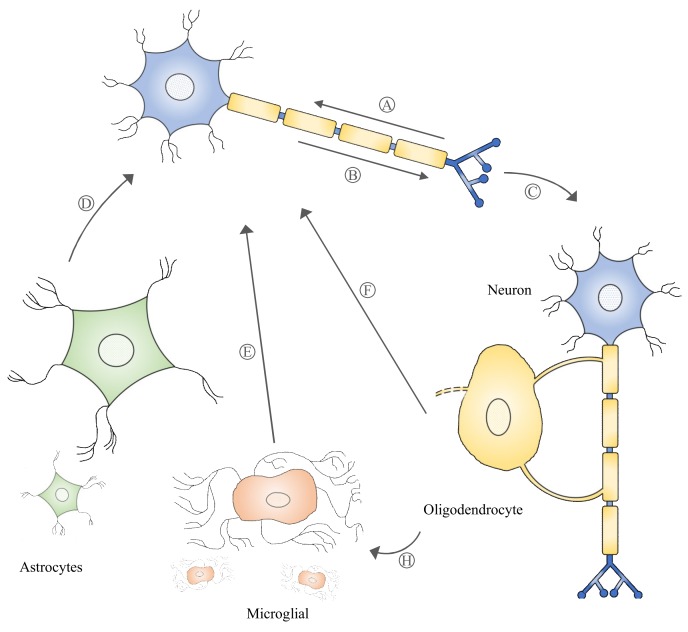

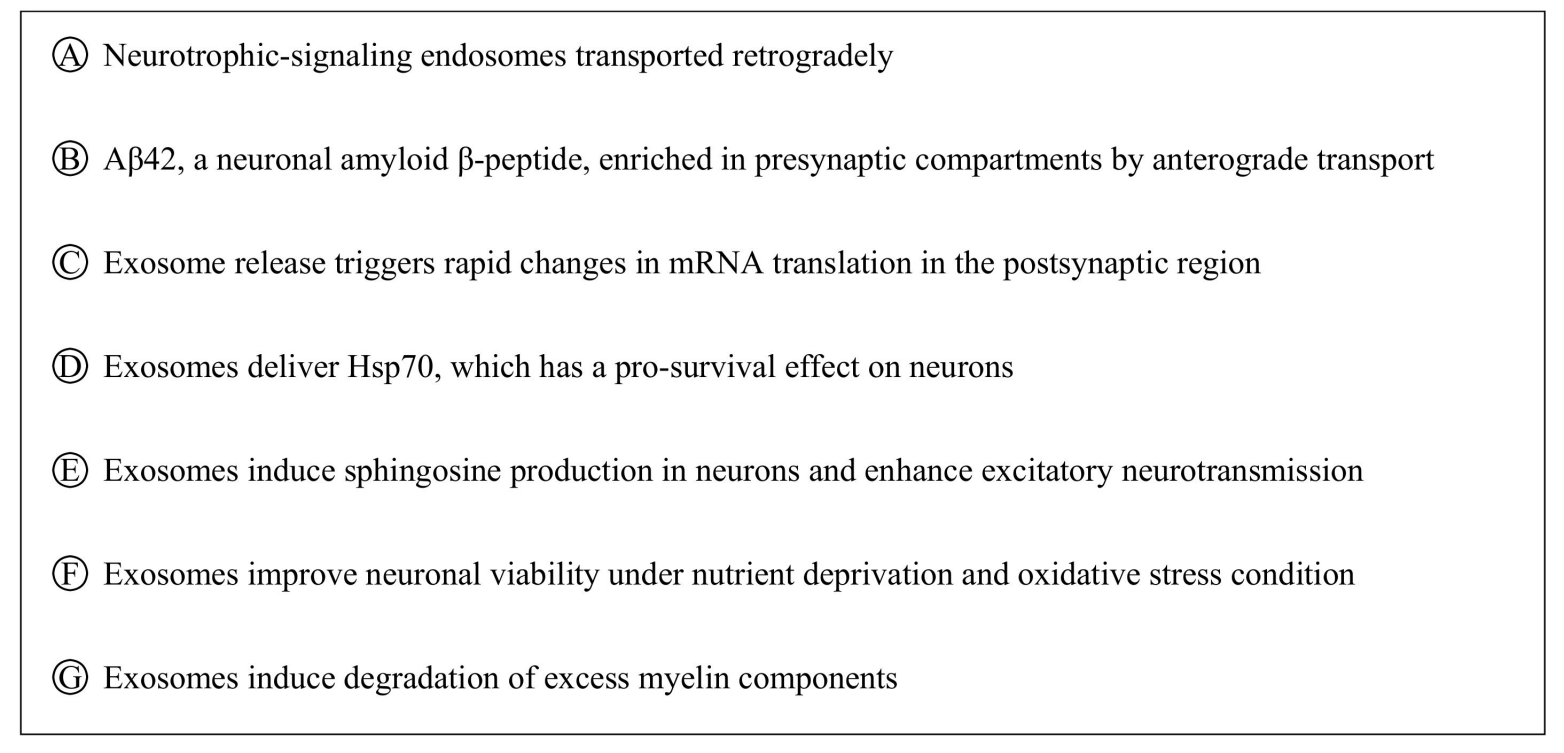
Multivesicular bodies can move retrogradely and anterogradely in neural cells (A-B), which may lead to their release into the extracellular space as exosomes. Exosomes participating in intercellular communication (signaling among neurons and/or non-neuronal cells, C–G) play a critical role in neuronal physiology and pathology.

Exosome biogenesis begins with the formation of multivesicular bodies (MVBs) within the endosomal compartment of the cell. The formation of MVBs is driven by endosomal sorting complexes required for transport (ESCRT) machinery, which sort and package molecules by incorporating them into vesicles. The fusion of MVBs with lysosomes results in the degradation of the encapsulated materials. Meanwhile, MVBs that are directed to fuse with the cell plasma membrane are secreted extracellularly as exosomes. Evidence of MVBs within neurons was observed under an electron microscope while studying the structure of neurons [31]. MVBs are differentially distributed within the neuronal compartments and many have been found to reside in the cell body and dendrites [32]. An in vitro study demonstrated that MVBs in neuronal cells harbor and transport neurotrophin signaling-endosomes retrogradely in the axon to the cell body [33]. Several studies [32,34] also provide evidence that MVBs can undergo anterograde axonal transport. A study revealed that Aβ42, a neuronal amyloid β-peptide, was absent from the postsynaptic compartments and was enriched only within MVBs in presynaptic compartments [35]. MVBs residing in synaptic boutons have been shown to be in close contact with the presynaptic membrane [36]. The ability of MVBs to be transported anterogradely or retrogradely, which ultimately leads to the release of exosomes extracellularly, suggests that exosomes participate in the bidirectional inter-neuronal transfer of information (Figure 1). Notably, exosomes released by neuronal cells were shown to cause rapid changes in translation of messenger RNAs (mRNAs) in the postsynaptic region to affect synaptic plasticity, suggesting its role in the neural plasticity-associated translational regulation [37]. Exosomes, as a novel means of inter-neuronal communication, may play critical roles in many physiological processes such as synapse growth and plasticity [38].

### 3.2. Neuronal and Non-Neuronal Cell Cross-Talk Mediated by Exosomes

Neurons and non-neuronal cells are parts of the nervous system that form a highly complex network in which each communicates with and complements the other to support neural network formation, organization, and functioning [39,40]. Exosomes have been described as a novel form of information exchange between cells with the capability to influence various cellular functions [29]. The ability of exosomes to regulate and influence a diverse range of biological processes within the nervous system has been reported [41,42]. Neuronal signals through neurotransmitter glutamate could regulate exosome release by oligodendrocytes [43]. It was discovered that the internalization of oligodendrocyte exosomes by neurons resulted in improved neuronal viability under nutrient deprivation and oxidative stress conditions [43]. Under thermal and oxidative stress, astrocytes were found to release exosomes harboring Hsp70, which has a pro-survival effect on neurons [44]. Microglia-derived exosome uptake by neurons has been shown to induce sphingosine production in neurons and enhance excitatory neurotransmission [45]. Interestingly, the uptake of oligodendrocyte exosomes by microglia did not trigger an inflammatory response, suggesting that these exosomes may be targeted to microglia for the degradation of excess myelin components [46]. These data suggest that exosomes released by local neurons and supporting cells such as oligodendrocytes, astrocytes, and microglia participate in intercellular communication (Figure 1) and that their role of supporting and maintaining neuronal homeostasis within the nervous system is critical to proper nerve cell, neural circuit, and nervous system function.

### 3.3. Exosomes’ Ability to Cross the Blood–Brain Barrier

The blood–brain barrier is a tightly regulated interface that protects the brain and maintains a homeostatic environment. Exosomes’ ability to breach the blood–brain barrier was implicated in a range of different pathophysiological processes. Exosomes released by the choroid plexus could cross the brain parenchyma and be taken up by microglia and astrocytes [47,48]. During systemic inflammation, choroid plexus-derived exosomes internalized by microglia and astrocytes could trigger miRNA target repression and induce the upregulation of inflammatory genes [47]. Moreover, in response to peripheral inflammation, cerebellar Purkinje neurons uptake of exosomes from the periphery displayed altered miRNA profiles [49]. A recent study on Parkinson’s disease revealed that exosomes secreted by human erythrocytes contained pathogenic α-synuclein, which co-localized with microglia and was observed in the cerebral nuclei, cortex, interbrain, midbrain, and substantia nigra [50,51]. The results suggested that peripheral insults provoking systemic inflammation could induce exosomes from the peripheral circulation to cross the blood–brain barrier [50,51]. Andras et al. also demonstrated the ability of the human immunodeficiency virus to increase the exosomal amyloid beta cargo and their release from brain endothelial cells to mediate the transfer of amyloid beta to astrocytes and pericytes, resulting in amyloid accumulation in the infected brain [52]. It is notable that under pathological conditions, peripheral exosomes contribute to the leakiness of the blood–brain barrier and, upon entry, can affect the brain’s biological activities [49,53,54].

## 4. Roles of Exosomes in the Nervous System

Exosomes are released constitutively or stimulus-dependently by numerous cell types within the nervous system [55]. Exosomes secreted by cells represent a means of cellular waste disposal or to mediate the intercellular exchange of their bioactive molecular cargo [29]. The fundamental molecular mechanisms of exosome biogenesis, and their release and uptake by targeted cells, are intended to ensure the proper functioning of the nervous system through optimal inter-neuronal communication and information processing [56]. On the other hand, the dysregulation of exosomes and their properties can lead to the development of a pathological state [56]. Exosomes engaged in a complex network of biological and pathological processes modulate and influence nervous system function including neuronal development, repair, maintenance and regeneration [55].

### 4.1. Exosomes in Neuroprotection

Exosomes with neuroprotective properties have been described as taking part in an important mechanism contributing to neuronal survival and function. A recent study revealed that astroglial released exosomes containing Apolipoprotein D (ApoD) have neuroprotective roles in neurons [57]. The investigators reported that the uptake of ApoD-exosomes by neuronal cells showed greater viability under oxidative stress, suggesting that ApoD-exosomes might promote functional integrity as well as the survival of neurons [57]. Xin et al. reported that miR-17-92 cluster-enriched exosomes could activate the PI3K/Akt/mTOR/GSK-3β signaling pathway and enhance neural plasticity and functional recovery in rodents with stroke [58]. Several authors [59,60,61,62] have also reported the impact of mesenchymal stem cell (MSC) exosomes on functional outcomes (e.g., cognitive and sensorimotor functions) in rodents with traumatic brain injury (TBI). MSC exosomes administered into rodent models of TBI revealed reduced cortical lesions, attenuated cellular apoptosis, and modulated neuroinflammation [59,60]. The outcomes of the neurological assessment of rodents with TBI demonstrated improved neurobehavioral performance and accelerated functional recovery [59,60]. Moreover, MSC exosomes’ ability to mediate neuronal survival and improved functionality (e.g., cognition and memory function) have been described in various neurological disorders including status epilepticus [63], Parkinson’s disease, and Alzheimer’s disease [64]. Exosome interactions with the nervous system are capable of inducing neurogenesis, promoting axonal and vascular remodeling, and modulating neuroinflammation, thereby mediating a neuroprotective mechanism and improved neurological outcomes.

### 4.2. Exosomes in Neurodegeneration

While exosomes’ role in neuroprotection is evident by the fact that they promote functional integrity and the survival of neurons, their involvement in neurodegeneration is attributed to their abilities to transfer pathogenic entities and promote the spread of diseases [65,66]. The pathogenesis underlying many neurodegenerative disorders (e.g., status epilepticus, Parkinson’s disease, and Alzheimer’s disease), both sporadic or genetically inherited forms, were consequences of molecular and cellular mechanisms that involve the abnormal misfolding and aggregation of proteins [67,68]. The abnormal accumulation and aggregation of disease-specific proteins could form intracellular inclusions or extracellular aggregates [68]. Amyloid beta and tau deposition in the brain has been identified as a toxic event by which exosome mediates to impair synaptic structure and function in Alzheimer’s disease [69], while the progressive accumulation of α-synuclein in the brain through exosomes has been linked to Parkinson’s disease [50,70]. Recent evidence has also implicated exosomes in aging processes [71]. Eitan et al. observed that exosome concentration in the plasma was found to decrease with age in humans and that the reduced exosome concentration in the circulation may be due, in part, to the increased internalization of exosomes by B cells, which were observed in the older individuals as compared to the younger individuals [72]. In a mouse study, Zhang et al. found a correlation between the age-dependent loss of hypothalamic neural stem/progenitor cells and aging-related physiological decline [73]. They also revealed that a concomitant decrease in exosomal miRNAs in the cerebrospinal fluid is associated with the loss of hypothalamic neural stem/progenitor cells, which led to accelerated aging in mice. However, a slowing of aging was observed when the mice were treated with exosomes from healthy hypothalamic neural stem/progenitor cells, suggesting that aging speed is substantially controlled by hypothalamic stem cells, partially through the release of exosomal miRNAs. Together, exosomes involved in the transport of pathogenic biomolecules (e.g., proteins and miRNAs) can contribute to the progression of neurodegenerative diseases. 

## 5. Exosomes and Clinical Symptoms Associated with Cancer and Its Therapy

Exosomes and their role in the evolution of cancer have expanded over the years. Many investigators have reported that oncogenic signaling molecules found in cancer exosomes were, in part, responsible for the pathogenesis and development of cancer [74]. Cancer cells also release exosomes that mediate therapy resistance, resulting in increased cancer cell survival and DNA repair [75]. In recent years, the impact of cancer and cancer therapies on neurobiological and behavioral changes (e.g., cachexia, fatigue, peripheral neuropathy, and cognitive impairment) is being recognized as an important clinical issue among cancer survivors. Several investigators [53,76,77,78] highlighted that changes in exosomal content and their release as a result of cancer and its therapy may influence neurobiological functioning and alter behavior.

### 5.1. Cancer Cachexia

Cancer cachexia is a condition that causes extreme weight loss and muscle wasting. The underlying mechanisms of the exosomes mediating cancer cachexia have recently been investigated. It was discovered that miRNAs and proteins in exosomes were responsible for the pathogenesis of the disease [79]. Several miRNAs (e.g., miR-155, miR-21, and miR-29a [80,81]) in exosomes were identified as orchestrating the molecular and biochemical disruptions observed in cachexia. The miRNAs transported in exosomes could contribute to muscle mass wasting by modulating inflammatory pathways and regulating protein synthesis and degradation pathways in the skeletal muscle [80,82]. In a functional study, Zhang et al. showed that tumor exosomes containing heat shock proteins (i.e., Hsp70 and Hsp90) could stimulate muscle catabolism through the activation toll-like receptor 4 on muscle cells, which further leads to the tumor induction of cachexia in mice [83]. Exosomes’ participation in promoting the catabolism of muscle cells and mediating skeletal muscle atrophy was observed as contributing to cancer cachexia.

### 5.2. Cancer-Related Fatigue

Cancer-related fatigue is another side effect of cancer and its treatment. It has been defined as a distressing, persistent, subjective sense of physical, emotional, and/or cognitive tiredness or exhaustion related to cancer and/or cancer treatment that is not proportional to recent activity and that interferes with usual functioning [84]. While the use of proteomics to understand cancer-related fatigue remains to be established [85], Minton et al. demonstrated differences in plasma protein expression in cancer-related fatigue syndrome compared to non-fatigued control subjects [86]. Nevertheless, Khalyfa et al. showed that chronic sleep fragmentation can alter exosomal miRNA cargo and affect the biological function (i.e., proliferative, migratory, and extravasation properties) of lung tumor cells in mice [87]. Similar results were also observed when human adenocarcinoma tumor cell lines were treated with plasma exosomes derived from patients with obstructive sleep apnea [87]. It was also reported that physical fatigue can be linked to the exosomal transfer of miRNAs affecting skeletal muscle function [88]. Although the etiology of cancer-related fatigue is poorly understood, studies revealed differences in their exosomal cargo content between a control group and a cancer-related fatigue group. Delineating the mechanisms that exosomes regulate may provide further insights to better understand exosomes’ role in cancer-related fatigue.

### 5.3. Peripheral Neuropathy

Peripheral neuropathy refers to damage of the peripheral nerves. Cancer and its treatment can cause peripheral neuropathy. Recently, a case control study by Chen et al. discovered that exosomes isolated from the serum of breast cancer patients receiving taxane treatment contained different subcellular and functional categories of proteins [89]. It was discovered that twelve protein signatures were associated with the development of taxane-induced peripheral neuropathy [89]. The study found that taxane-treated patients who developed severe neuropathy did not regain a homeostatic balance between inflammation and detoxification. It was suggested that patients identified as having low inflammatory and detoxification responses before taxane treatment may have a greater risk of developing taxane-induced peripheral neuropathy [89]. Although the link between exosomes and taxane-induced peripheral neuropathy has not been well-characterized, exosomes may play a role in mediating the homeostatic balance between inflammation and detoxification, which could be critical to reducing the risk of developing peripheral neuropathy in cancer patients undergoing taxane treatment.

While cancer survivors also suffer from cognitive dysfunction, it has been reported that the development of CRCI is a multifactorial process [90]. In addition to cancer and cancer treatment, numerous other factors such as cancer cachexia [91] and cancer-related fatigue [90] participate in the process of influencing CRCI. The role of exosomes and CRCI will be further discussed in the next section. The effort to unravel the functional role of exosomes and their cargo content can provide important insight into the mechanisms by which exosomes mediate.

## 6. Association of Exosomes and CRCI

As it is increasingly recognized that cognitive dysfunction is a complication of cancer and its treatment, numerous studies have provided evidence that multiple domains of cognition such as memory, concentration, and executive function can be seen affected in patients with CRCI [9,91]. To harmonize the study of cognitive function in patients with cancer, the International Cognitive and Cancer Task Force (ICCTF) developed recommendations focusing on key dimensions of cognition (i.e., learning, memory, executive function, and processing speed) [7,92]. The recommended tests for assessing these various functions include the Hopkins Verbal Learning Test–Revised (for learning and memory), Trail Making Test (for executive function and processing speed), and Controlled Oral Word Association Test (for executive function). In addition, novel approaches including the incorporating of neuroimaging (e.g., magnetic resonance imaging scan) and basic/translational science (e.g., biomarker studies and preclinical animal models) would facilitate a better understanding of the pathophysiology of CRCI [92]. In recent years, several studies have provided preliminary evidence, and have suggested the existence of a relationship between exosomes and CRCI. Here, we review the literature by which exosomes affect cognition as a result of cancer and its treatment.

### 6.1. Cancer Exosomes and Cognitive Impairment

Exosomes released by cancer cells of the central nervous system (CNS) (e.g., brain tumor) or non-CNS (e.g., cancer that spreads from the periphery to the brain) can contribute to reduced cognition by modulating the cellular mechanism that perturbs brain function. Certain cancers (e.g., glioblastoma [78,93] and medulloblastoma [76,94]) arising from the CNS have been shown to express exosomes that could affect cellular integrity. For example, glioblastoma was shown to remodel the tumor microenvironment and lead to chemotherapy resistance (e.g., temozolomide) through the intracellular transfer of oncogenic long non-coding RNA SBF2-AS1 by exosomes [93]. In addition to the ability to achieve chemoresistance, glioblastoma secretion of pro-permeability factor (e.g., Semaphorin3A) through exosomes has been associated with the loss of endothelial barrier integrity, resulting in debilitating cognitive deficits [78].

Exosomes released by non-CNS cancer cells can play a significant role in cancer progression and metastasis. It has been observed that cancer cells discard the tumor suppressor miRNA, such as miR23b through exosomes to acquire metastatic properties [95]. Moreover, a variety of cancers including lung cancer, breast cancer, and melanoma can lead to brain metastasis [96]. Kuroda et al. reported that CD46, integrin α5, or integrin αV receptors in blood–brain barrier endothelial cells are involved in the internalization of exosomes derived from the brain-metastatic melanoma cell [97]. In addition to brain metastasis, melanoma is frequently associated with CNS complications including cognitive impairment [97,98]. While it remains to be determined, cancer complications and the loss of blood–brain barrier integrity might be exacerbated by exosomes secreted by cancer cells, such as melanoma exosomes [97].

Cancer cells were also found to secrete exosomes that enact on other mechanisms (e.g., interfere with cytokine-mediated immune-activation pathways), which mediates immune modulation [99]. Tumor-derived exosomes were shown to regulate the function of immune cells including macrophages, T-cells, and natural killer cells [100]. It has been discovered that breast cancer-derived exosomes containing a high level of annexin A2 induced the expression of vascular endothelial growth factor receptor 1 and matrix metalloproteinase 9 in lung and brain sections [101]. The study also found that high annexin A2 in exosomes triggers macrophage-mediated activation of the p38 mitogen-activated protein kinases, nuclear factor-kappa B pathways, and the signal transducer and activator of transcription 3 pathways, as well as increased pro-inflammatory cytokines [101]. It is plausible that exosomes released by cancer cells could interact with immune cells to affect distant organs (e.g., lungs and brain) and their function.

Exosomes released by cancer cells of CNS may act locally and modulate the cellular mechanism to affect brain activity and influence cognitive function directly. Meanwhile, exosomes released by non-CNS cancer cells may induce impaired cognitive function in several ways. This includes their ability to activate brain metastasis in cancer cells that compromises blood–brain barrier integrity, to harbor a pro-permeability factor that induces the permeability of the blood–brain barrier, and/or to trigger the stress response in the brain through a peripheral immune challenge by immune cells directed by cancer exosomes.

### 6.2. Cancer-Therapy-Derived Exosomes and Cognitive Impairment

The use of radiation therapy in the treatment of a variety of CNS-related malignancies can affect brain structure and function, leading to cognitive deterioration [102]. Radiation used in cancer therapy can also activate the biogenesis and induce exosome secretion from cancer cells [103]. Hinzman et al. highlighted that several molecules (i.e., triglycerides, platelet activating factor, carnitine, and C-16 sphinganine) involved in inflammation were enriched in the exosomes of mice exposed to cranial ionizing radiation [104]. In addition, the exosomes of irradiated mice expressed a higher level of CD63 and were morphologically different from those of sham-irradiated mice [104]. Although radiation therapy has a significant effect on cognitive functioning, a few studies have demonstrated the potential use of exosomes derived from neural stem cells to ameliorate radiation-induced cognitive dysfunction [105,106].

### 6.3. Mechanisms Associated with Exosomes and CRCI

**Figure 2:**
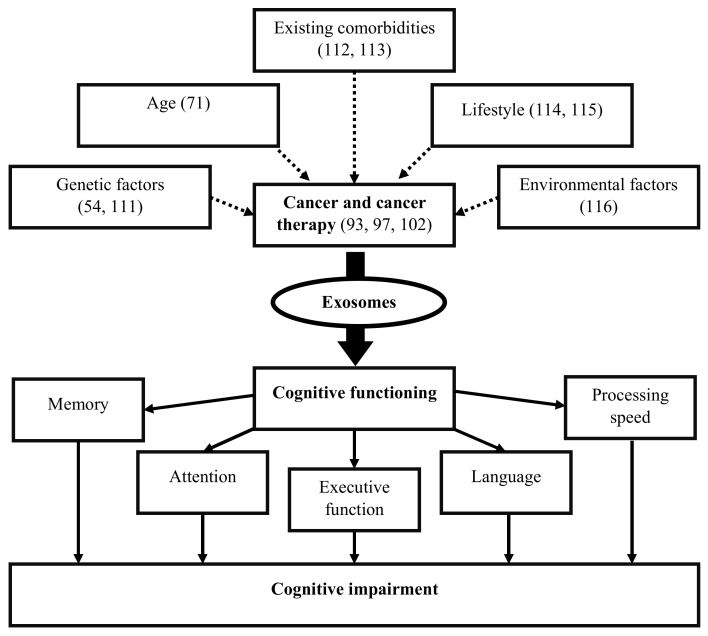
Exosomes release as a result of cancer and cancer therapy may alter cognitive functions, resulting in cognitive impairment in cancer survivors. Genetics, age, existing comorbidities, lifestyle, and environmental factors predisposed to (dotted lines) cancer and cancer therapy may affect exosome biogenesis, its cargo content, function, and activity to influence cognitive functioning including memory, attention, executive function, language, and processing speed.

**Figure 3:**
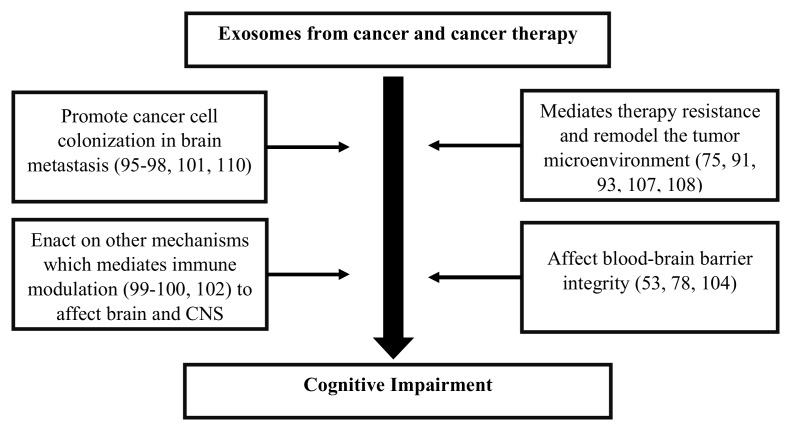
Flow diagram describing the potential role of exosomes and their ability to influence the microenvironment of the brain and central nervous system (CNS), which causes cognitive impairment in cancer survivors.

Exosomes released by cells could serve as a means to discard undesired biomolecules or mediate a variety of paracrine signaling mechanisms. Exosomes could protect their cargoes from degradation (e.g., enzymatic cleavage) and overcome immune surveillance during their transit through the extracellular microenvironment. Cancer cells have been found to remove tumor suppressor miRNAs through exosomes in order to promote metastasis [95]. Cancer cells also release exosomes to promote tumorigenic phenotypes, which includes growth, invasion, and immunosuppression [107]. Exosomes released by cancer cells that accumulate in the brain could induce blood–brain barrier permeability [53], and their translocation into brain tissue may elicit functional changes in behavior and cognition [91]. While cancer therapies adversely impact cognition, they also affect exosome biogenesis and release causing changes to the anatomy of the brain [104]. Cancer therapies also cause cellular stress that affect the release and composition of exosomes, thereby impacting neighboring and distant cells (e.g., brain) [108]. Moreover, radio- and chemotherapy may also influence the development of treatment-resistant cancer cells [75], possibly through cellular reprogramming [109] and/or the formation of brain metastasis [110]. Genetics [54,111], age [71], existing comorbidities [112,113], lifestyle [114,115], and environmental [116] factors are likely to alter the extracellular fate of exosomes, which leads to CRCI (Figure 2). Changes in exosomal cargo content and their release may further influence diverse mechanisms/pathways capable of impacting the brain and CNS function, which could result in CRCI (Figure 3).

## 7. Potential Implication of Exosomes in CRCI Research

Exosomes play a part in fundamental physiological processes, such as neuronal communication, but also participate in pathological conditions, such as cancer and chemotherapy-induced cognitive impairment. Although the precise mechanisms underlying CRCI remain to be established, several studies suggest there is a link between exosomes and CRCI. Exosome biogenesis, its cargo content, function, and activity may be influenced by cancer [97], cancer therapies [93,102], genetics [54,111], age [71], existing comorbidities [112,113], lifestyle [114,115], and environmental [116] factors. Differences in exosomal content (e.g., miRNAs and proteins) and release may exert protective or damaging effects that could alter the brain and CNS function, and cause declining cognitive health in cancer survivors. Unraveling the mechanisms and pathways influenced by exosomes is critical to understand the relationship between exosomes, insults (e.g., cancer and cancer therapy) and the neurocognitive network in the brain [117]. Advancing our knowledge on exosomes and neurocognition is critical to develop exosome-based therapeutics. The action of stem cell-derived exosomes within the irradiated microenvironment of the brain was shown to have neuroprotective properties, and was found to reduce radiation-induced pathology and cognitive dysfunction [106]. The potential uses of exosomes derived from stem cells might serve as a promising strategy to ameliorate cognitive dysfunction in patients suffering from CRCI.

## 8. Conclusions

Exosomes are small cell-derived vesicles, initially thought to function as a means of cellular waste disposal, but later discovered to be critical mediators of intercellular signaling. Exosome biogenesis and composition depends on cellular origin and homeostatic state. Exosomes are multifunctional, acting and functioning differently under physiological and pathological conditions. The ability of exosomes to cross the blood–brain barrier, which enables bidirectional exchange of biomolecules, indicates exosomes may serve as potential biomarkers of CNS health and disease. In this review, we summarize the role of exosomes and their ability to influence the microenvironment of the brain, resulting in CRCI. Investigations of cell-type specific exosomes and their cargo are necessary to delineate novel mechanisms/pathways and better understand how exosomes impact CNS function and CRCI. Advancing our knowledge in the biology of exosomes will be critical for the future development of exosome-based therapeutics for CRCI.

## Abbreviations

ApoD	Apolipoprotein D
CNS	Central nervous system
CRCI	Cancer-related cognitive impairment
ESCRT	Endosomal sorting complexes required for transport
ICCTF	International Cognitive and Cancer Task Force
MSC	Mesenchymal stem cell
MVBs	Multivesicular bodies
TBI	Traumatic brain injury

## References

[1] Fouad Y.A., Aanei C. (2017). Revisiting the hallmarks of cancer. Am. J. Cancer Res..

[2] Sellers W.R., Fisher D.E. (1999). Apoptosis and cancer drug targeting. J. Clin. Investig..

[3] Pistritto G., Trisciuoglio D., Ceci C., Garufi A., D’Orazi G. (2016). Apoptosis as anticancer mechanism: Function and dysfunction of its modulators and targeted therapeutic strategies. Aging.

[4] Hall E.J. (2019). Radiobiology for the Radiologist.

[5] Cheung Y.T., Ng T., Shwe M., Ho H.K., Foo K.M., Cham M.T., Lee J.A., Fan G., Tan Y.P., Yong W.S. (2015). Association of proinflammatory cytokines and chemotherapy-associated cognitive impairment in breast cancer patients: A multi-centered, prospective, cohort study. Ann. Oncol..

[6] Ng T., Dorajoo S.R., Cheung Y.T., Lam Y.C., Yeo H.L., Shwe M., Gan Y.X., Foo K.M., Loh W.K., Koo S.L. (2018). Distinct and heterogeneous trajectories of self-perceived cognitive impairment among Asian breast cancer survivors. Psychooncology.

[7] Wefel J.S., Vardy J., Ahles T., Schagen S.B. (2011). International Cognition and Cancer Task Force recommendations to harmonise studies of cognitive function in patients with cancer. Lancet Oncol..

[8] Chan R.J., McCarthy A.L., Devenish J., Sullivan K.A., Chan A. (2015). Systematic review of pharmacologic and non-pharmacologic interventions to manage cognitive alterations after chemotherapy for breast cancer. Eur. J. Cancer.

[9] Hardy S.J., Krull K.R., Wefel J.S., Janelsins M. (2018). Cognitive Changes in Cancer Survivors. Am. Soc. Clin. Oncol. Educ. Book.

[10] Janelsins M.C., Kesler S.R., Ahles T.A., Morrow G.R. (2014). Prevalence, mechanisms, and management of cancer-related cognitive impairment. Int. Rev. Psychiatry.

[11] Cheung Y.T., Shwe M., Tan Y.P., Fan G., Ng R., Chan A. (2012). Cognitive changes in multiethnic Asian breast cancer patients: A focus group study. Ann. Oncol..

[12] Correa D.D., Satagopan J., Baser R.E., Cheung K., Richards E., Lin M., Karimi S., Lyo J., DeAngelis L.M., Orlow I. (2014). APOE polymorphisms and cognitive functions in patients with brain tumors. Neurology.

[13] Tan C.J., Lim S.W.T., Toh Y.L., Ng T., Yeo A., Shwe M., Foo K.M., Chu P., Jain A., Koo S.L. (2019). Replication and Meta-analysis of the Association between BDNF Val66Met Polymorphism and Cognitive Impairment in Patients Receiving Chemotherapy. Mol. Neurobiol..

[14] Pendergrass J.C., Targum S.D., Harrison J.E. (2018). Cognitive Impairment Associated with Cancer: A Brief Review. Innov. Clin. Neurosci..

[15] De Toro J., Herschlik L., Waldner C., Mongini C. (2015). Emerging roles of exosomes in normal and pathological conditions: New insights for diagnosis and therapeutic applications. Front. Immunol..

[16] Mayo S.J., Kuruvilla J., Laister R.C., Ayala A.P., Alm M., Byker W., Kelly D.L., Saligan L. (2018). Blood-based biomarkers of cancer-related cognitive impairment in non-central nervous system cancer: Protocol for a scoping review. BMJ Open.

[17] Wefel J.S., Kesler S.R., Noll K.R., Schagen S.B. (2015). Clinical characteristics, pathophysiology, and management of noncentral nervous system cancer-related cognitive impairment in adults. CA Cancer J. Clin..

[18] Han L., Lam E.W., Sun Y. (2019). Extracellular vesicles in the tumor microenvironment: Old stories, but new tales. Mol. Cancer.

[19] Zhang X., Yuan X., Shi H., Wu L., Qian H., Xu W. (2015). Exosomes in cancer: Small particle, big player. J. Hematol. Oncol..

[20] Kobayashi M., Rice G.E., Tapia J., Mitchell M., Salomon C. (2015). Exosomes are fingerprints of originating cells: Potential biomarkers for ovarian cancer. Res. Rep. Biochem..

[21] Isola A.L., Chen S. (2017). Exosomes: The Messengers of Health and Disease. Curr. Neuropharmacol..

[22] Rajagopal C., Harikumar K.B. (2018). The Origin and Functions of Exosomes in Cancer. Front. Oncol..

[23] Lee Y., El Andaloussi S., Wood M.J. (2012). Exosomes and microvesicles: Extracellular vesicles for genetic information transfer and gene therapy. Hum. Mol. Genet..

[24] Leavitt R.J., Limoli C.L., Baulch J.E. (2019). miRNA-based therapeutic potential of stem cell-derived extracellular vesicles: A safe cell-free treatment to ameliorate radiation-induced brain injury. Int. J. Radiat. Biol..

[25] Witwer K.W., Buzas E.I., Bemis L.T., Bora A., Lasser C., Lotvall J., Nolte-’t Hoen E.N., Piper M.G., Sivaraman S., Skog J. (2013). Standardization of sample collection, isolation and analysis methods in extracellular vesicle research. J. Extracell. Vesicles.

[26] Lotvall J., Hill A.F., Hochberg F., Buzas E.I., Di Vizio D., Gardiner C., Gho Y.S., Kurochkin I.V., Mathivanan S., Quesenberry P. (2014). Minimal experimental requirements for definition of extracellular vesicles and their functions: A position statement from the International Society for Extracellular Vesicles. J. Extracell. Vesicles.

[27] Thery C., Witwer K.W., Aikawa E., Alcaraz M.J., Anderson J.D., Andriantsitohaina R., Antoniou A., Arab T., Archer F., Atkin-Smith G.K. (2018). Minimal information for studies of extracellular vesicles 2018 (MISEV2018): A position statement of the International Society for Extracellular Vesicles and update of the MISEV2014 guidelines. J. Extracell. Vesicles.

[28] Pereda A.E. (2014). Electrical synapses and their functional interactions with chemical synapses. Nat. Rev. Neurosci..

[29] Zhang G., Yang P. (2018). A novel cell-cell communication mechanism in the nervous system: Exosomes. J. Neurosci. Res..

[30] Chivet M., Hemming F., Pernet-Gallay K., Fraboulet S., Sadoul R. (2012). Emerging role of neuronal exosomes in the central nervous system. Front. Physiol..

[31] Palay S.L., Palade G.E. (1955). The fine structure of neurons. J. Biophys. Biochem. Cytol..

[32] Altick A.L., Baryshnikova L.M., Vu T.Q., von Bartheld C.S. (2009). Quantitative analysis of multivesicular bodies (MVBs) in the hypoglossal nerve: Evidence that neurotrophic factors do not use MVBs for retrograde axonal transport. J. Comp. Neurol..

[33] Parton R.G., Simons K., Dotti C.G. (1992). Axonal and dendritic endocytic pathways in cultured neurons. J. Cell Biol..

[34] Korkut C., Li Y., Koles K., Brewer C., Ashley J., Yoshihara M., Budnik V. (2013). Regulation of postsynaptic retrograde signaling by presynaptic exosome release. Neuron.

[35] Yu Y., Jans D.C., Winblad B., Tjernberg L.O., Schedin-Weiss S. (2018). Neuronal Abeta42 is enriched in small vesicles at the presynaptic side of synapses. Life Sci. Alliance.

[36] Koles K., Nunnari J., Korkut C., Barria R., Brewer C., Li Y., Leszyk J., Zhang B., Budnik V. (2012). Mechanism of evenness interrupted (Evi)-exosome release at synaptic boutons. J. Biol. Chem..

[37] Goldie B.J., Dun M.D., Lin M., Smith N.D., Verrills N.M., Dayas C.V., Cairns M.J. (2014). Activity-associated miRNA are packaged in Map1b-enriched exosomes released from depolarized neurons. Nucleic Acids Res..

[38] Lachenal G., Pernet-Gallay K., Chivet M., Hemming F.J., Belly A., Bodon G., Blot B., Haase G., Goldberg Y., Sadoul R. (2011). Release of exosomes from differentiated neurons and its regulation by synaptic glutamatergic activity. Mol. Cell. Neurosci..

[39] Stogsdill J.A., Eroglu C. (2017). The interplay between neurons and glia in synapse development and plasticity. Curr. Opin. Neurobiol..

[40] Jakel S., Dimou L. (2017). Glial Cells and Their Function in the Adult Brain: A Journey through the History of Their Ablation. Front. Cell. Neurosci..

[41] Janas A.M., Sapon K., Janas T., Stowell M.H., Janas T. (2016). Exosomes and other extracellular vesicles in neural cells and neurodegenerative diseases. Biochim. Biophys. Acta.

[42] Sharma P., Mesci P., Carromeu C., McClatchy D.R., Schiapparelli L., Yates J.R., Muotri A.R., Cline H.T. (2019). Exosomes regulate neurogenesis and circuit assembly. Proc. Natl. Acad. Sci. USA.

[43] Fruhbeis C., Frohlich D., Kuo W.P., Amphornrat J., Thilemann S., Saab A.S., Kirchhoff F., Mobius W., Goebbels S., Nave K.A. (2013). Neurotransmitter-triggered transfer of exosomes mediates oligodendrocyte-neuron communication. PLoS Biol..

[44] Taylor A.R., Robinson M.B., Gifondorwa D.J., Tytell M., Milligan C.E. (2007). Regulation of heat shock protein 70 release in astrocytes: Role of signaling kinases. Dev. Neurobiol..

[45] Antonucci F., Turola E., Riganti L., Caleo M., Gabrielli M., Perrotta C., Novellino L., Clementi E., Giussani P., Viani P. (2012). Microvesicles released from microglia stimulate synaptic activity via enhanced sphingolipid metabolism. EMBO J..

[46] Fitzner D., Schnaars M., van Rossum D., Krishnamoorthy G., Dibaj P., Bakhti M., Regen T., Hanisch U.K., Simons M. (2011). Selective transfer of exosomes from oligodendrocytes to microglia by macropinocytosis. J. Cell Sci..

[47] Balusu S., Van Wonterghem E., De Rycke R., Raemdonck K., Stremersch S., Gevaert K., Brkic M., Demeestere D., Vanhooren V., Hendrix A. (2016). Identification of a novel mechanism of blood-brain communication during peripheral inflammation via choroid plexus-derived extracellular vesicles. EMBO Mol. Med..

[48] Grapp M., Wrede A., Schweizer M., Huwel S., Galla H.J., Snaidero N., Simons M., Buckers J., Low P.S., Urlaub H. (2013). Choroid plexus transcytosis and exosome shuttling deliver folate into brain parenchyma. Nat. Commun..

[49] Ridder K., Keller S., Dams M., Rupp A.K., Schlaudraff J., Del Turco D., Starmann J., Macas J., Karpova D., Devraj K. (2014). Extracellular vesicle-mediated transfer of genetic information between the hematopoietic system and the brain in response to inflammation. PLoS Biol..

[50] Matsumoto J., Stewart T., Sheng L., Li N., Bullock K., Song N., Shi M., Banks W.A., Zhang J. (2017). Transmission of alpha-synuclein-containing erythrocyte-derived extracellular vesicles across the blood-brain barrier via adsorptive mediated transcytosis: Another mechanism for initiation and progression of Parkinson’s disease?. Acta Neuropathol. Commun..

[51] Matsumoto J., Stewart T., Banks W.A., Zhang J. (2017). The Transport Mechanism of Extracellular Vesicles at the Blood-Brain Barrier. Curr. Pharm. Des..

[52] Andras I.E., Leda A., Contreras M.G., Bertrand L., Park M., Skowronska M., Toborek M. (2017). Extracellular vesicles of the blood-brain barrier: Role in the HIV-1 associated amyloid beta pathology. Mol. Cell. Neurosci..

[53] Tominaga N., Kosaka N., Ono M., Katsuda T., Yoshioka Y., Tamura K., Lotvall J., Nakagama H., Ochiya T. (2015). Brain metastatic cancer cells release microRNA-181c-containing extracellular vesicles capable of destructing blood-brain barrier. Nat. Commun..

[54] Riva P., Battaglia C., Venturin M. (2019). Emerging Role of Genetic Alterations Affecting Exosome Biology in Neurodegenerative Diseases. Int. J. Mol. Sci..

[55] Yuyama K., Igarashi Y. (2016). Physiological and pathological roles of exosomes in the nervous system. Biomol. Concepts.

[56] Chivet M., Javalet C., Hemming F., Pernet-Gallay K., Laulagnier K., Fraboulet S., Sadoul R. (2013). Exosomes as a novel way of interneuronal communication. Biochem. Soc. Trans..

[57] Pascua-Maestro R., Gonzalez E., Lillo C., Ganfornina M.D., Falcon-Perez J.M., Sanchez D. (2018). Extracellular Vesicles Secreted by Astroglial Cells Transport Apolipoprotein D to Neurons and Mediate Neuronal Survival Upon Oxidative Stress. Front. Cell. Neurosci..

[58] Xin H., Katakowski M., Wang F., Qian J.Y., Liu X.S., Ali M.M., Buller B., Zhang Z.G., Chopp M. (2017). MicroRNA cluster miR-17-92 Cluster in Exosomes Enhance Neuroplasticity and Functional Recovery After Stroke in Rats. Stroke.

[59] Ni H., Yang S., Siaw-Debrah F., Hu J., Wu K., He Z., Yang J., Pan S., Lin X., Ye H. (2019). Exosomes Derived From Bone Mesenchymal Stem Cells Ameliorate Early Inflammatory Responses Following Traumatic Brain Injury. Front. Neurosci..

[60] Zhang Y., Chopp M., Meng Y., Katakowski M., Xin H., Mahmood A., Xiong Y. (2015). Effect of exosomes derived from multipluripotent mesenchymal stromal cells on functional recovery and neurovascular plasticity in rats after traumatic brain injury. J. Neurosurg..

[61] Kim D.K., Nishida H., An S.Y., Shetty A.K., Bartosh T.J., Prockop D.J. (2016). Chromatographically isolated CD63+CD81+ extracellular vesicles from mesenchymal stromal cells rescue cognitive impairments after TBI. Proc. Natl. Acad. Sci. USA.

[62] Zhang Y., Chopp M., Zhang Z.G., Katakowski M., Xin H., Qu C., Ali M., Mahmood A., Xiong Y. (2017). Systemic administration of cell-free exosomes generated by human bone marrow derived mesenchymal stem cells cultured under 2D and 3D conditions improves functional recovery in rats after traumatic brain injury. Neurochem. Int..

[63] Long Q., Upadhya D., Hattiangady B., Kim D.K., An S.Y., Shuai B., Prockop D.J., Shetty A.K. (2017). Intranasal MSC-derived A1-exosomes ease inflammation, and prevent abnormal neurogenesis and memory dysfunction after status epilepticus. Proc. Natl. Acad. Sci. USA.

[64] Cui G.H., Guo H.D., Li H., Zhai Y., Gong Z.B., Wu J., Liu J.S., Dong Y.R., Hou S.X., Liu J.R. (2019). RVG-modified exosomes derived from mesenchymal stem cells rescue memory deficits by regulating inflammatory responses in a mouse model of Alzheimer’s disease. Immun. Ageing.

[65] Jan A.T., Malik M.A., Rahman S., Yeo H.R., Lee E.J., Abdullah T.S., Choi I. (2017). Perspective Insights of Exosomes in Neurodegenerative Diseases: A Critical Appraisal. Front. Aging Neurosci..

[66] Kalani A., Tyagi A., Tyagi N. (2014). Exosomes: Mediators of neurodegeneration, neuroprotection and therapeutics. Mol. Neurobiol..

[67] Takalo M., Salminen A., Soininen H., Hiltunen M., Haapasalo A. (2013). Protein aggregation and degradation mechanisms in neurodegenerative diseases. Am. J. Neurodegener. Dis..

[68] Howitt J., Hill A.F. (2016). Exosomes in the Pathology of Neurodegenerative Diseases. J. Biol. Chem..

[69] Sardar Sinha M., Ansell-Schultz A., Civitelli L., Hildesjo C., Larsson M., Lannfelt L., Ingelsson M., Hallbeck M. (2018). Alzheimer’s disease pathology propagation by exosomes containing toxic amyloid-beta oligomers. Acta Neuropathol..

[70] Koh Y.H., Tan L.Y., Ng S.Y. (2018). Patient-Derived Induced Pluripotent Stem Cells and Organoids for Modeling Alpha Synuclein Propagation in Parkinson’s Disease. Front. Cell. Neurosci..

[71] D’Anca M., Fenoglio C., Serpente M., Arosio B., Cesari M., Scarpini E.A., Galimberti D. (2019). Exosome Determinants of Physiological Aging and Age-Related Neurodegenerative Diseases. Front. Aging Neurosci..

[72] Eitan E., Green J., Bodogai M., Mode N.A., Baek R., Jorgensen M.M., Freeman D.W., Witwer K.W., Zonderman A.B., Biragyn A. (2017). Age-Related Changes in Plasma Extracellular Vesicle Characteristics and Internalization by Leukocytes. Sci. Rep..

[73] Zhang Y., Kim M.S., Jia B., Yan J., Zuniga-Hertz J.P., Han C., Cai D. (2017). Hypothalamic stem cells control ageing speed partly through exosomal miRNAs. Nature.

[74] Mashouri L., Yousefi H., Aref A.R., Ahadi A.M., Molaei F., Alahari S.K. (2019). Exosomes: Composition, biogenesis, and mechanisms in cancer metastasis and drug resistance. Mol. Cancer.

[75] Steinbichler T.B., Dudas J., Skvortsov S., Ganswindt U., Riechelmann H., Skvortsova I.-I. (2019). Therapy resistance mediated by exosomes. Mol. Cancer.

[76] Ciregia F., Urbani A., Palmisano G. (2017). Extracellular Vesicles in Brain Tumors and Neurodegenerative Diseases. Front. Mol. Neurosci..

[77] Rajendran L., Bali J., Barr M.M., Court F.A., Kramer-Albers E.M., Picou F., Raposo G., van der Vos K.E., van Niel G., Wang J. (2014). Emerging roles of extracellular vesicles in the nervous system. J. Neurosci..

[78] Treps L., Edmond S., Harford-Wright E., Galan-Moya E.M., Schmitt A., Azzi S., Citerne A., Bidere N., Ricard D., Gavard J. (2016). Extracellular vesicle-transported Semaphorin3A promotes vascular permeability in glioblastoma. Oncogene.

[79] Chitti S.V., Fonseka P., Mathivanan S. (2018). Emerging role of extracellular vesicles in mediating cancer cachexia. Biochem. Soc. Trans..

[80] Wu Q., Sun S., Li Z., Yang Q., Li B., Zhu S., Wang L., Wu J., Yuan J., Wang C. (2019). Breast cancer-released exosomes trigger cancer-associated cachexia to promote tumor progression. Adipocyte.

[81] He W.A., Calore F., Londhe P., Canella A., Guttridge D.C., Croce C.M. (2014). Microvesicles containing miRNAs promote muscle cell death in cancer cachexia via TLR7. Proc. Natl. Acad. Sci. USA.

[82] Marinho R., Alcantara P.S.M., Ottoch J.P., Seelaender M. (2017). Role of Exosomal MicroRNAs and myomiRs in the Development of Cancer Cachexia-Associated Muscle Wasting. Front. Nutr..

[83] Zhang G., Liu Z., Ding H., Zhou Y., Doan H.A., Sin K.W.T., Zhu Z.J., Flores R., Wen Y., Gong X. (2017). Tumor induces muscle wasting in mice through releasing extracellular Hsp70 and Hsp90. Nat. Commun..

[84] Bower J.E. (2014). Cancer-related fatigue--mechanisms, risk factors, and treatments. Nat. Rev. Clin. Oncol..

[85] Minton O., Stone P.C. (2010). Review: The use of proteomics as a research methodology for studying cancer-related fatigue: A review. Palliat. Med..

[86] Minton O., Stone P.C. (2013). The identification of plasma proteins associated with cancer-related fatigue syndrome (CRFS) in disease-free breast cancer patients using proteomic analysis. J. Pain Symptom Manag..

[87] Khalyfa A., Almendros I., Gileles-Hillel A., Akbarpour M., Trzepizur W., Mokhlesi B., Huang L., Andrade J., Farre R., Gozal D. (2016). Circulating exosomes potentiate tumor malignant properties in a mouse model of chronic sleep fragmentation. Oncotarget.

[88] Aoi W., Sakuma K. (2014). Does regulation of skeletal muscle function involve circulating microRNAs?. Front. Physiol..

[89] Chen E.I., Crew K.D., Trivedi M., Awad D., Maurer M., Kalinsky K., Koller A., Patel P., Kim Kim J., Hershman D.L. (2015). Identifying Predictors of Taxane-Induced Peripheral Neuropathy Using Mass Spectrometry-Based Proteomics Technology. PLoS ONE.

[90] Pesendorfer L.M., Zimmer P., Galvao D., Zopf E.M., Bloch W., Baumann F.T. (2016). Impact of physical inactivity on the multifactorial process of developing cancer-related cognitive impairments. J. Cancer Sci. Ther..

[91] Olson B., Marks D.L. (2019). Pretreatment Cancer-Related Cognitive Impairment-Mechanisms and Outlook. Cancers.

[92] Deprez S., Kesler S.R., Saykin A.J., Silverman D.H.S., de Ruiter M.B., McDonald B.C. (2018). International Cognition and Cancer Task Force Recommendations for Neuroimaging Methods in the Study of Cognitive Impairment in Non-CNS Cancer Patients. J. Natl. Cancer Inst..

[93] Zhang Z., Yin J., Lu C., Wei Y., Zeng A., You Y. (2019). Exosomal transfer of long non-coding RNA SBF2-AS1 enhances chemoresistance to temozolomide in glioblastoma. J. Exp. Clin. Cancer Res..

[94] Epple L.M., Griffiths S.G., Dechkovskaia A.M., Dusto N.L., White J., Ouellette R.J., Anchordoquy T.J., Bemis L.T., Graner M.W. (2012). Medulloblastoma exosome proteomics yield functional roles for extracellular vesicles. PLoS ONE.

[95] Ostenfeld M.S., Jeppesen D.K., Laurberg J.R., Boysen A.T., Bramsen J.B., Primdal-Bengtson B., Hendrix A., Lamy P., Dagnaes-Hansen F., Rasmussen M.H. (2014). Cellular disposal of miR23b by RAB27-dependent exosome release is linked to acquisition of metastatic properties. Cancer Res..

[96] Fecci P.E., Champion C.D., Hoj J., McKernan C.M., Goodwin C.R., Kirkpatrick J.P., Anders C.K., Pendergast A.M., Sampson J.H. (2019). The Evolving Modern Management of Brain Metastasis. Clin. Cancer Res..

[97] Kuroda H., Tachikawa M., Yagi Y., Umetsu M., Nurdin A., Miyauchi E., Watanabe M., Uchida Y., Terasaki T. (2019). Cluster of Differentiation 46 Is the Major Receptor in Human Blood-Brain Barrier Endothelial Cells for Uptake of Exosomes Derived from Brain-Metastatic Melanoma Cells (SK-Mel-28). Mol. Pharm..

[98] Tawbi H.A., Boutros C., Kok D., Robert C., McArthur G. (2018). New Era in the Management of Melanoma Brain Metastases. Am. Soc. Clin. Oncol. Educ. Book.

[99] Barros F.M., Carneiro F., Machado J.C., Melo S.A. (2018). Exosomes and Immune Response in Cancer: Friends or Foes?. Front. Immunol..

[100] Othman N., Jamal R., Abu N. (2019). Cancer-Derived Exosomes as Effectors of Key Inflammation-Related Players. Front. Immunol..

[101] Maji S., Chaudhary P., Akopova I., Nguyen P.M., Hare R.J., Gryczynski I., Vishwanatha J.K. (2017). Exosomal Annexin II Promotes Angiogenesis and Breast Cancer Metastasis. Mol. Cancer Res..

[102] Lumniczky K., Szatmari T., Safrany G. (2017). Ionizing Radiation-Induced Immune and Inflammatory Reactions in the Brain. Front. Immunol..

[103] Jabbari N., Nawaz M., Rezaie J. (2019). Ionizing Radiation Increases the Activity of Exosomal Secretory Pathway in MCF-7 Human Breast Cancer Cells: A Possible Way to Communicate Resistance against Radiotherapy. Int. J. Mol. Sci..

[104] Hinzman C.P., Baulch J.E., Mehta K.Y., Girgis M., Bansal S., Gill K., Li Y., Limoli C.L., Cheema A.K. (2019). Plasma-derived extracellular vesicles yield predictive markers of cranial irradiation exposure in mice. Sci. Rep..

[105] Baulch J.E., Acharya M.M., Allen B.D., Ru N., Chmielewski N.N., Martirosian V., Giedzinski E., Syage A., Park A.L., Benke S.N. (2016). Cranial grafting of stem cell-derived microvesicles improves cognition and reduces neuropathology in the irradiated brain. Proc. Natl. Acad. Sci. USA.

[106] Smith S.M., Giedzinski E., Angulo M.C., Lui T., Lu C., Park A.L., Tang S., Martirosian V., Ru N., Chmielewski N.N. (2019). Functional Equivalence of Stem Cell and Stem Cell-Derived Extracellular Vesicle Transplantation to Repair the Irradiated Brain. Stem Cells Transl. Med..

[107] Rahbarghazi R., Jabbari N., Sani N.A., Asghari R., Salimi L., Kalashani S.A., Feghhi M., Etemadi T., Akbariazar E., Mahmoudi M. (2019). Tumor-derived extracellular vesicles: Reliable tools for Cancer diagnosis and clinical applications. Cell Commun. Signal..

[108] Ni J., Bucci J., Malouf D., Knox M., Graham P., Li Y. (2019). Exosomes in Cancer Radioresistance. Front. Oncol..

[109] Vlashi E., Pajonk F. (2015). The metabolic state of cancer stem cells-a valid target for cancer therapy?. Free Radic. Biol. Med..

[110] Morad G., Moses M.A. (2019). Brainwashed by extracellular vesicles: The role of extracellular vesicles in primary and metastatic brain tumour microenvironment. J. Extracell. Vesicles.

[111] Egan M.F., Kojima M., Callicott J.H., Goldberg T.E., Kolachana B.S., Bertolino A., Zaitsev E., Gold B., Goldman D., Dean M. (2003). The BDNF val66met polymorphism affects activity-dependent secretion of BDNF and human memory and hippocampal function. Cell.

[112] Kodidela S., Gerth K., Haque S., Gong Y., Ismael S., Singh A., Tauheed I., Kumar S. (2019). Extracellular Vesicles: A Possible Link between HIV and Alzheimer’s Disease-Like Pathology in HIV Subjects?. Cells.

[113] Saeedi S., Israel S., Nagy C., Turecki G. (2019). The emerging role of exosomes in mental disorders. Transl. Psychiatry.

[114] Suire C.N., Eitan E., Shaffer N.C., Tian Q., Studenski S., Mattson M.P., Kapogiannis D. (2017). Walking speed decline in older adults is associated with elevated pro-BDNF in plasma extracellular vesicles. Exp. Gerontol..

[115] Crenshaw B.J., Kumar S., Bell C.R., Jones L.B., Williams S.D., Saldanha S.N., Joshi S., Sahu R., Sims B., Matthews Q.L. (2019). Alcohol Modulates the Biogenesis and Composition of Microglia-Derived Exosomes. Biology.

[116] Harischandra D.S., Ghaisas S., Rokad D., Kanthasamy A.G. (2017). Exosomes in Toxicology: Relevance to Chemical Exposure and Pathogenesis of Environmentally Linked Diseases. Toxicol. Sci..

[117] Bressler S.L., Tognoli E. (2006). Operational principles of neurocognitive networks. Int. J. Psychophysiol..

